# Caressed by music: Related preferences for velocity of touch and tempo of music?

**DOI:** 10.3389/fpsyg.2023.1135988

**Published:** 2023-03-02

**Authors:** Uta Sailer, Manuela Zucknick, Bruno Laeng

**Affiliations:** ^1^Department of Behavioural Medicine, Faculty of Medicine, Institute of Basic Medical Sciences, University of Oslo, Oslo, Norway; ^2^Department of Biostatistics, Faculty of Medicine, Institute of Basic Medical Sciences, University of Oslo, Oslo, Norway; ^3^Department of Psychology, University of Oslo, Oslo, Norway; ^4^RITMO Centre for Interdisciplinary Studies in Rhythm, Time and Motion, University of Oslo, Oslo, Norway

**Keywords:** CT-fibers, entrainment, multisensory, supramodal, preference, pleasant touch

## Abstract

Given that both hearing and touch are ‘mechanical senses’ that respond to physical pressure or mechanical energy and that individuals appear to have a characteristic internal or spontaneous tempo, individual preferences in musical and touch rhythms might be related. We explored this in two experiments probing individual preferences for tempo in the tactile and auditory modalities. Study 1 collected ratings of received stroking on the forearm and measured the velocity the participants used for stroking a fur. Music tempo preferences were assessed as mean beats per minute of individually selected music pieces and via the adjustment of experimenter-selected music to a preferred tempo. Heart rate was recorded to measure levels of physiological arousal. We found that the preferred tempo of favorite (self-selected) music correlated positively with the velocity with which each individual liked to be touched. In Study 2, participants rated videos of repeated touch on someone else’s arm and videos of a drummer playing with brushes on a snare drum, both at a variety of tempos. We found that participants with similar rating patterns for the different stroking speeds did not show similar rating patterns for the different music beats. The results suggest that there may be a correspondence between preferences for favorite music and felt touch, but this is either weak or it cannot be evoked effectively with vicarious touch and/or mere drum beats. Thus, if preferences for touch and music are related, this is likely to be dependent on the specific type of stimulation.

## Introduction

Individuals appear to have idiosyncratic preferences for the speed or tempo of music. When asking subjects what metronome tempo is optimal, i.e., neither too slow nor too fast, most responses lay in the range between 500 and 700 ms (Fraisse, 1982), corresponding to 86–120 beats per minute (bpm). Such a preferred musical tempo is also subject to wide inter-individual variation (e.g., Fraisse, 1982; Bauer et al., 2015). Thus, individuals tend to exhibit a *spontaneous perceptual tempo* (SPT) or rate in a series of sounds, but also for pulsating light, which feels “just right” (Fraisse, 1982).

Tempo preferences also exist in the tactile modality, where there seem to be idiosyncratic and fairly stable tempo preferences for the touch of the skin by another person. On a group level, participants typically rate stroking touch velocities between 1 and 10 cm/s as the most pleasant (e.g., Löken et al., 2009; Ackerley et al., 2014; Sehlstedt et al., 2016; Sailer and Ackerley, 2019). Stroking at these velocities maximally activates a specific class of nerve fibers in our hairy skin, the so-called CT fibers (Vallbo et al., 1993, 1999; Ackerley et al., 2014), which are assumed to play an important role in interpersonal touch. As with musical tempo, there are also large differences between individuals in their preferred stroking tempo (Croy et al., 2020).

Individual preferences are also present in the tempo of motor behaviors- William Stern (1900) is credited not only for coining the concept of Intelligence Quotient (IQ) but also of individual differences in “mental tempo,” e.g., the preferred *spontaneous motor tempo* (SMT; McAuley, 2010). Other research confirmed that individuals differ in their characteristic “internal” or “spontaneous” tempo, both in behaviors like movement and drawing, but also in cognition and the preferences for the rate of sensory stimulation (e.g., Rimoldi, 1951; McPherson et al., 2018). These preferences are similar not in terms of absolute isochrony, but of relative coupling of stimulation speeds.

### Relationships between preferences in different modalities and with the motor system

Such preferences do not only exist independently from each other, but are related across modalitiesand with the motor system. The psychiatrist Stein (1977) observed that mania, which is characterized by a generally fast pace of behavior and decision-making, is also associated with a preference for a fast tempo of music in such patients. In healthy participants, SMT and SPT tend to have comparable rates in the same individual (McAuley, 2010). Based on a strong and positive correlation (of about 0.75) between them, it was argued that SMT and SPT have a common psychological (or physiological) basis (McAuley et al., 2006). Individual preferences in musical tempo have also been linked to preferred SMT for tapping, which appears to be around 100–120 bpm (Moelants, 2002; Levitin et al., 2018). The mentioned strong link between preferred motor and listening tempo, found by McAuley and colleagues (McAuley et al., 2006), also suggests that preferred motor and auditory tempo are coupled.

The idea that bodily motion and music are deeply connected has been discussed since the ancient Greeks (Todd, 1995). For example, Plato had already observed that mothers sing lullabies while simultaneously rocking their children (Schoen-Nazzaro, 1978). Indeed, humans tend to spontaneously synchronize their body movements with music from infancy (Zentner and Eerola, 2010), and the universal feature of relaxing, slow, movements of lullabies across cultures (Mehr et al., 2019) may serve the purpose to soothe and decelerate bodily motion. People tend to tap (typically with a foot) along with perceived pulse (Drake et al., 2000) and change their walking speed when listening to music (Styns et al., 2007). In current musicological accounts, music is thought to be “embodied,” meaning that bodily processes like motor activity are part-and-parcel of the experience of music (Juntunen and Hyvönen, 2004; Maes, 2016), both when listening, dancing to it, and in music making. The musical experience of ‘groove’ further indicates a tendency to move together with the music, plausibly in an attempt to understand through the body rhythmic complexities (Koelsch et al., 2019; Spiech et al., 2022). Coupling between patterns of speed and tempo, often also in synchrony, is common between motor executions of bodily movements and perceived patterns (e.g., stepping in time), and among perceptual processes (Todd and Lee, 2015). In many conditions, tactile synchronization (e.g., finger tapping; Ammirante et al., 2016) closely matches auditory sequences. The intertwinement of music and motion *via* the concept of space has also been stressed by Di Stefano (2022). In general, it has been suggested that “all human performance can be evaluated within a rhythmic framework” (Jones, 1976, p. 340).

In the present study, we want to focus on similarities in preferences for touch and music.

### What is behind the relationship between these preferences?

#### Common properties of the different sensory systems

Certain dimensions of sensory experiences are not specific to any one sensory modality but instead are common to multiple sensory modalities (see f.ex. the notion of “unity of the senses,” Marks, 1978). These “amodal” or supra-sensory properties may include features such as spatial location, temporal duration, brightness and intensity, which can be perceived through multiple senses, such as vision, hearing, and touch (von Hornbostel, 1931; Boring, 1942; Marks et al., 1986).

Along these lines, tactile information acquired from touching a sound source is not intrinsically different form the physical (vibratory) energy used by the auditory system (Ammirante et al., 2016). Both hearing and touch are ‘mechanical senses’ that respond to physical pressure or mechanical energy, although each *via* specialized receptor systems. In fact, the receptor systems for touch and audition share some unique properties. For example, perceived stimulus magnitude (i.e., loudness in the case of sound, and intensity in the case of vibration) is enhanced by the presentation of a prior stimulus with similar frequency (Zwislocki et al., 1974; Zwislocki and Sokolich, 1974; Verrillo and Gescheider, 1975; Gescheider et al., 1977). Thus, both auditory and tactile receptors can integrate the neural responses of two stimuli. At higher levels of processing, the similarities between the two systems are preserved. For example, in the mouse somatosensory cortex, vibration frequency is encoded in a similar manner to how sound pitch is represented in the auditory cortex (Prsa et al., 2019). That is, the somatosensory neurons appear to be tuned to a particular combination of frequency and amplitude which corresponded to what the mouse perceived (i.e., similarly to auditory neurons where the perceived pitch changes with frequency and loudness).

Related to this finding in mice, there is evidence for common neural circuits for frequency processing in audition and touch in humans (Butler et al., 2012). In a different study, participants were faster to detect stimuli that were presented both in the auditory and somatosensory modality than if they were presented only in one modality (Murray et al., 2005). Neuroimaging indicated that these multisensory interactions occurred at a very early stage of sensory processing (Murray et al., 2005), which supports the idea that auditory and tactile processing might be based on common neural mechanisms. In line with this, a common neural network was activated when detecting rhythmic sequences or beats presented in the auditory and tactile modality (Araneda et al., 2017).

#### Cross-modal perception and integration

Cross-modal perception means that the perception of a stimulus by one sense influences the perception of a stimulus by another sense. Humans tend to confuse different stimuli in touch and audition when they are presented simultaneously or in alternation (von Békésy, 1959). Several examples for cross-modal perception of tactile and auditory stimuli have been reported in the literature (for reviews, see Di Stefano and Spence, 2022; Spence, 2022). The perception of roughness in surfaces is influenced by touch-produced sounds (Jousmaki and Hari, 1998; Yau et al., 2009). Sounds unrelated to the touch itself, namely background music, also influenced the perceived softness of fabrics (Imschloss and Kuehnl, 2019). Not only roughness, but also harmony has been suggested to be analogously perceived in touch and audition (Spence and Di Stefano, 2022). The perceived “sexiness” of music was even able to increase the sexiness of touch performed by a machine (Fritz et al., 2017).

In addition to cross-modal perception, there is also evidence for multisensory integration of touch and audition. Multisensory integration means that different senses provide a person with information about the same perceptual object, e.g., when one both hears and feels a prickly branch brushing across the skin. Supporting the notion that tactile input is involved in “feeling” musical rhythm, it was found that musical meter can be perceived through the activation of touch afferents, and that meter information from audition and touch was integrated into a common percept (Huang et al., 2012). Possibly supporting such an integration process, touch vibration activates human auditory cortex (Caetano and Jousmäki, 2006; Schurmann et al., 2006), and auditory frequency is in turn represented in somatosensory cortex (Perez-Bellido et al., 2018). The interrelationship between the sense of touch and hearing also forms the basis for various applications, e.g., to improve the listening experience of music (Turchet et al., 2021; Yoo et al., 2014).

In sum, given the interrelationships in ratings of roughness, softness, harmony and sexiness of tactile and auditory stimuli, preferences for the tempo of stimuli in these two modalities may also be related.

#### Pacemaker

A correspondence of preferences in speed for stimuli in different modalities could be the expression of some “central” timing process or pattern generator. Such a pattern generator could be in the form of an “inner pacemaker” at a level that is above that of specific effectors or group muscles or of specific sensory modalities that has its own pace, but can also entrain to external rhythms. It could be fundamental to behaviors that need to be performed at a controlled pace or rhythm (e.g., walking, feeding, breathing, swimming, etc.), but it may also provide a pace to our attention and conscious perception (Chakravarthi and VanRullen, 2012; Fiebelkorn and Kastner, 2019), possibly by synchronizing patterns of cortical activity (Yuste et al., 2005; VanRullen et al., 2007; Pfurtscheller et al., 2017, 2018), including during music listening (Large and Jones, 1999; Large et al., 2015). Most likely, as several studies in physiology indicate (Katz, 2016), there could be several neural pattern generators, each able to generate periodic activity with a specific function. These pattern generaters may also be ‘coupled’ to one another, and some more strongly than others, due to evolutionary constraints or histories in the development of different motor and sensory organs in a species (Ito et al., 2022).

If a supramodal pattern generator (SPG) couples perceptions across multiple modalities, one would expect to observe regularities between patterns of speed and tempo across the sensory modalities. These correspondences may not necessarily be expressed as absolute synchrony in phase or oscillation (since each sensory system has its inherently different rate of processing), but they may be related in terms of their relative speed or pace, e.g., a tendency to be in the relative slow or fast range for each modality. Indeed, one expectation of the present study is that an individual’s internal tempo may generate a pulse that drives all the other modality-specific pattern generators. If so, a key prediction is that if an individual’s internal tempo ticks fast, this would be coupled to a fast pace across all sensory modalities, though within their own range of pacing, whereas if it ticks slow, this would be matched by slow paces across all sensory modalities.

It has been suggested that the heart beat may have such a pacemaker function (Palser et al., 2021). Several scholars have considered the possibility that the heart beat might influence tempo preferences for either the rhythms of touch or music or both (North and Hargreaves, 2008; Parncutt, 2009). Mothers tend to stroke their babies in a velocity related to their own heart rate (Van Puyvelde et al., 2019; Bytomski et al., 2020). Physical arousal caused by different forms of exercise has been found to be positively associated with preferred musical tempo (e.g., Karageorghis and Jones, 2014). In a previous study where participants controlled the musical tempo themselves in order to find their favorite musical tempo, adjustments tended to be close to one’s own heart beat (Iwanaga, 1995). Hence, heart rate may also influence the association between touch and music.

### The present study

This study aimed to explore correspondences in relative preferred speed and tempo of perceptual events in different modalities: pleasant touch and pleasant sounds (music). Specifically, we expected an individual’s preferred touch velocity or tempo (when tactile stimulation is repeated consistently) to be related to the same individual’s preferred musical tempo or beat of favorite musical pieces. Furthermore, we also tested whether each of these preferences is related to individual heart rate.

In Study 1, the preferred touch velocity was assessed both in an active mode, where participants freely chose the velocity for stroking a material, and in a passive mode, where participants were stroked at different velocities and rated touch pleasantness. Similarly, preferences for musical tempo were tested in an active mode, where the participants actively adjusted the beat of experimenter-selected music pieces to their own preferred tempo, and a passive mode, by determining the beat of favorite self-selected music. We also investigated ongoing heart rhythm changes, namely heart rate (HR) – which is associated with arousal (e.g., Lacey and Lacey, 1974; Lang et al., 1993) – and heart rate variability (HRV), which reflects changes and adaptation of the nervous system (Shaffer et al., 2014). This was done in order to explore whether heart rate measures influence the potential association between preferences in touch and music.

Study 2 was an online study comparing the preferred stroking velocity in videos and the preferred beat of a drummer playing a rhythm in different tempos. In this case, the tactile stimulation was only simulated as the participants observed the forearm of another person unknown to them being stroked, but they did not experience the stroking on their own arm.

The videos of the drummer had normal soundtrack so that the musical rhythm stimulation was in this case both auditory and visual.

## Study 1: Preference in speed of being caressed and implicit preferences for musical tempo

We decided to assess implicit preferences for musical tempo by asking participants to provide several examples of preferred musical pieces. This approach was openly exploratory, since several features besides rhythm (e.g., melodic lines, the timbre of musical instruments) clearly contribute to musical appreciation. However, if rhythm is a core dimension of musical esthetics, we would deem likely that highly appreciated musical pieces tend to reflect an individual’s preference for musical tempo. Note that no explicit mention was made about their tempo or any other musical features, and preferences were extracted implicitly by calculating the central tendency in tempo of the self-selected musical examples.

Preferences for speed of caressing were measured directly by ratings of tactile stimulation in the laboratory. Our expectations were that the preferred tempos of touch and music would be related and, specifically, that the preferred velocity for passive touch would correlate positively with the tempo of self-selected music, as both measures assess preferences for passively received stimuli (i.e., music is “given” to us, who are necessarily its passive recipients, since we have limited attentive control that we can exert over listening to music being played). Similarly, active touch was expected to correlate positively with the adjusted tempo of experimenter-selected music, as both measures assess an actively chosen velocity.

We also expected that passive and active measures of touch would be related positively, but not necessarily to be identical or equally related to music preferences since we assume that our relation to music listening is more passive than active. Another expectation was that heart rate would correlate positively with velocities of preferred touch (active touch velocity) and the adjusted tempo of experimenter-selected music. Finally, we predicted that heart rate variability would be higher after having been touched, as found in previous studies (e.g., Triscoli et al., 2017a). We also explored whether relationships between the preferences for music and touch tempo could be mediated by body awareness (as probed by a questionnaire).

### Materials and methods

#### Participants

Students at the Psychology and Medical Departments at the University of Oslo were recruited *via* announcements and word of mouth. They received a voucher of 200 NOK (*ca.* 22 EUR) for participation. The study was approved by the ethical committee at the Dept. of Psychology, University of Oslo.

The study included 50 participants aged 19 to 33 years (*M =* 24.96, SD *=* 3.79), and 70% were female. None of the participants had acute skin diseases on the arm or any pain conditions. The majority (96%) of our participants were university students, and 54% of them were studying psychology (52% of the total sample). Five participants (10% of the total sample) were currently taking medications, four of which were antidepressants, and one was treated for attention-deficit/hyperactivity disorder.

#### Procedure

Prior to the experiment, the participants provided titles and the Internet links to five music pieces that they particularly liked to listen to in daily life. No other instructions were given. The obtained sound files were analyzed afterwards for their temporal properties, as described below.

**Figure 1:**
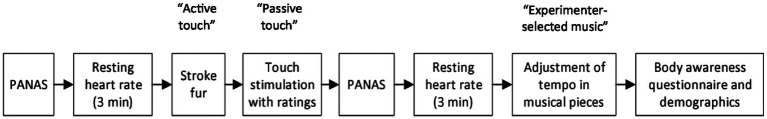
Order of tasks and measures collected during lab visit in study 1.

We invited participants to the laboratory at the Department of Behavioural Medicine at the University of Oslo. Upon arrival, each participant first completed a short mood questionnaire (PANAS; Watson et al., 1988; see Figure 1). After that, the first heart rate measurement was recorded over an interval of 3 min with three Ag/AgCl electrodes placed inferior to the right and left clavicle and the left 8th rib. Data were sampled at 1,000 kHz using a BioPac MP150 Nomadix wireless system (BioPac Systems Inc., Santa Barbara, CA, United States) and recorded with AcqKnowledge software (version 4.4, BioPac Systems Inc., CA, United States).

The participants were then seated at a table with their left arm resting on a pillow that was inflated to stabilize their arm. A warm electric heating pad made of sheep fur was placed on the table in front of the patient. Participants were asked to stroke this material “as if they were stroking a person close to them.” The time needed to complete 10 back-and-forth strokes across a fixed distance of 10 cm was recorded, and the mean speed of one stroke was used in the subsequent analysis.

Next, participants were stroked by use of a paintbrush made of soft goat hair attached to a robotic tactile stimulator (RTS, Dancer Design). Specifically, an area of 10 cm length was marked on the participant’s left, dorsal, forearm. Stroking was applied in a proximal-distal direction, with a pressure of 0.4 N. Twelve stroking velocities (0.3, 1, 2, 3, 4, 5, 6, 7, 8, 9, 10, and 30 cm/s); as in Luong et al. (2017) were presented three times each in pseudo-randomized order. After each stroke, the participants rated each of the velocities on two consecutive visual analog scales asking, “How did you experience the touch?” with the endpoints “pleasant” and “unpleasant” (coded as +10 and −10) and “How would you rate the velocity of the touch?” with the endpoints “too slow” and “too fast” (coded as −10 and + 10).

Following this passive touch task, participants filled out the PANAS questionnaire again and the heart rate was recorded over another 3-min interval. Next, they were presented with three pieces of music and one “practice piece” to begin with to become familiar with the procedure. The pieces were chosen in a way that made it unlikely that the participants were highly familiar with them. The three musical pieces were “Self-Image” by Co-Pilot,^1^ “Eight hundred Streets by Feet” by Esbjörn Svensson Trio, and “Bar Rumba” by Mo′Horizons, and they were always presented in this order. Participants were then asked to adjust the music’s tempo to a velocity that they found optimal. They had 3 min per music piece to determine the subjective optimal speed. Velocity was adjusted using the software Amazing Slow Downer 3.4.4 (Roni Music), which allows changing the speed of music without changing the pitch. Participants told the experimenter when they had decided on an optimal tempo, and the experimenter noted the percentage of change in tempo. The participants filled also out a questionnaire on body awareness (BAQ; Shields et al., 1989), because associations between different modalities might be higher in participants with high body awareness. Furthermore, participants answered demographic questions about age, sex, profession, and psychiatric medication. They were also asked whether they were familiar with the music pieces presented during the tempo adjustment task. All but one participant reported that they had not heard the music pieces before. This participant was also included in the analyses.

#### Measures

##### Touch measures

###### Passive touch velocity

We first averaged the participant’s pleasantness ratings for each velocity of passive touch over all three trials. In the second step, the most pleasant velocity (the one with the highest mean pleasantness rating) was identified individually for each participant. For two participants two velocities received the same highest rating so that no single favorite velocity could be determined. Thus, analyses on passive touch velocity included 48 participants.

###### Active touch velocity

“Active touch velocity” was calculated as the time needed to perform one average stroking movement across a fixed distance of 10 cm on the warm sheep fur.

##### Music measures

###### Tempo of self-selected music

The beats per minute (bpm) of the five preferred music pieces provided by the participants were determined using MixMeister BPM Analyzer (1.0). The standard deviation of the five music pieces per participant was determined, after which the bpms of these five music pieces were averaged per participant to obtain a central tendency estimate of preferred tempo that we labelled the “preferred tempo of self-selected music.” This variable was ‘winsorized’ (Ghosh and Vogt, 2012), as the data contained a data point that was more than 1.5 times the value of the interquartile range beyond the quartiles and therefore, an outlier. All subsequent analyses involving the preferred tempo of self-selected music were performed with the winsorized variable.

###### Tempo of experimenter-selected music

We calculated the mean percentage of speed change from the original tempo across all three presented music pieces for each participant and transformed this into the resulting preferred speed (in bpm) as a measure of “preferred tempo of experimenter-selected music.”

##### Physiological measures

###### Heart rate

For heart rate and HRV analysis, peak-to-peak R-R intervals were identified using the BioPac event-related analysis routine. All data were visually checked and corrected for missing heartbeats or multiple peak identifications. In the rare case of missing peaks, the R-peak was manually inserted into the correct position as recommended by the Task Force of the European Society of Cardiology and the North American Society of Pacing and Electrophysiology (“Heart rate variability: standards of measurement, physiological interpretation and clinical use. Task Force of the European Society of Cardiology and the North American Society of Pacing and Electrophysiology,” 1996). The R-R intervals were imported into Kubios software (Tarvainen et al., 2014), and the mean heart rate and HRV measures were determined. Mean heart rate was calculated separately for each ECG session resulting in the variables *HR1* and *HR2*.

Due to technical difficulties, no ECG data were available for two participants in the first ECG trial or for three participants in the second trial. Thus, analyses of HR1 included 48 participants, and analyses on HR2 47.

###### Heart rate variability

HRV was calculated as the root mean square of successive differences (RMSSD) between heartbeats. RMSSD seems to be a valid index of vagally mediated processes (Shaffer et al., 2014). HRV was determined for both ECG sessions, resulting in the variables HRV1 (*N* = 48) and HRV2 (*N* = 47).

##### Questionnaires

###### PANAS (positive and negative affect schedule)

The PANAS (Watson et al., 1988) is a 20-item self-report measure that is one of the most widely used scales in psychological and medical research to measure mood. It compromises two mood scales, one measuring positive affect and the other measuring negative affect. From those two scales, we calculated a *PANAS positive* and *PANAS negative* value by summing up item ratings for each measurement time point (giving the variables *PANAS1/2 positive/negative*).

###### BAQ (body awareness questionnaire)

The BAQ (Shields et al., 1989) is an 18-item self-report questionnaire that measures attentiveness to bodily processes (e.g., “I notice distinct body reactions when I am fatigued”). The BAQ includes items about bodily cycles and rhythms and the ability to detect small changes in normal physiological functioning. The BAQ scores are summed up (to give the variable *BAQ sum*), with low sum scores indicating low body awareness and high scores indicating high body awareness. One participant’s questionnaire failed to be registered. Therefore, analyses including the BAQ were performed with 49 participants.

#### Statistical analysis

All data were analyzed with SPSS for Windows (IBM, version 24). In order to determine whether the preferred tempos in touch and music are similar, several correlation analyses were conducted. All correlations involving passive touch were analyzed using Spearman correlations because this measure was scaled ordinally (Bortz, 2013). Pearson correlations were used for all other measures.

The relationship between heart rate and preferred velocities of touch as well as the preferred bpm for active music was investigated with further correlation analyses (two-tailed, Spearman for passive touch and Pearson for all other measures). Each touch or music measure was related to the heart rate measurement that occurred closest in time. Thus, HR1 was correlated with passive and active touch, whereas HR2 was correlated to active music. Altogether, eight different correlations were calculated. To adjust for multiple testing, the Benjamini-Hochberg procedure (Benjamini and Hochberg, 1995) was applied with a false discovery rate (FDR) of 0.25.

To determine whether mean heart rate, HRV, and/or mood changed during the experiment, HR1 and HR2, HRV1 and HRV2, and PANAS1 and PANAS2 were compared to each other using paired-samples t-tests with an FDR of 0.25.

To explore whether observed associations between different modalities were influenced by body awareness, significant correlations were followed up with mediation analysis using Hayes’ PROCESS macro (Hayes and Little, 2013). Passive touch was entered as the predictor variable, body awareness (BAQ sum score) as the mediator, and self-selected music as the criterion/outcome variable. Products were mean-centered and percentile bootstrap confidence intervals were used as these were found to be more robust in small samples with potential outliers (Creedon and Hayes, 2015).

### Results

#### Preferred velocities for touch

**Figure 2:**
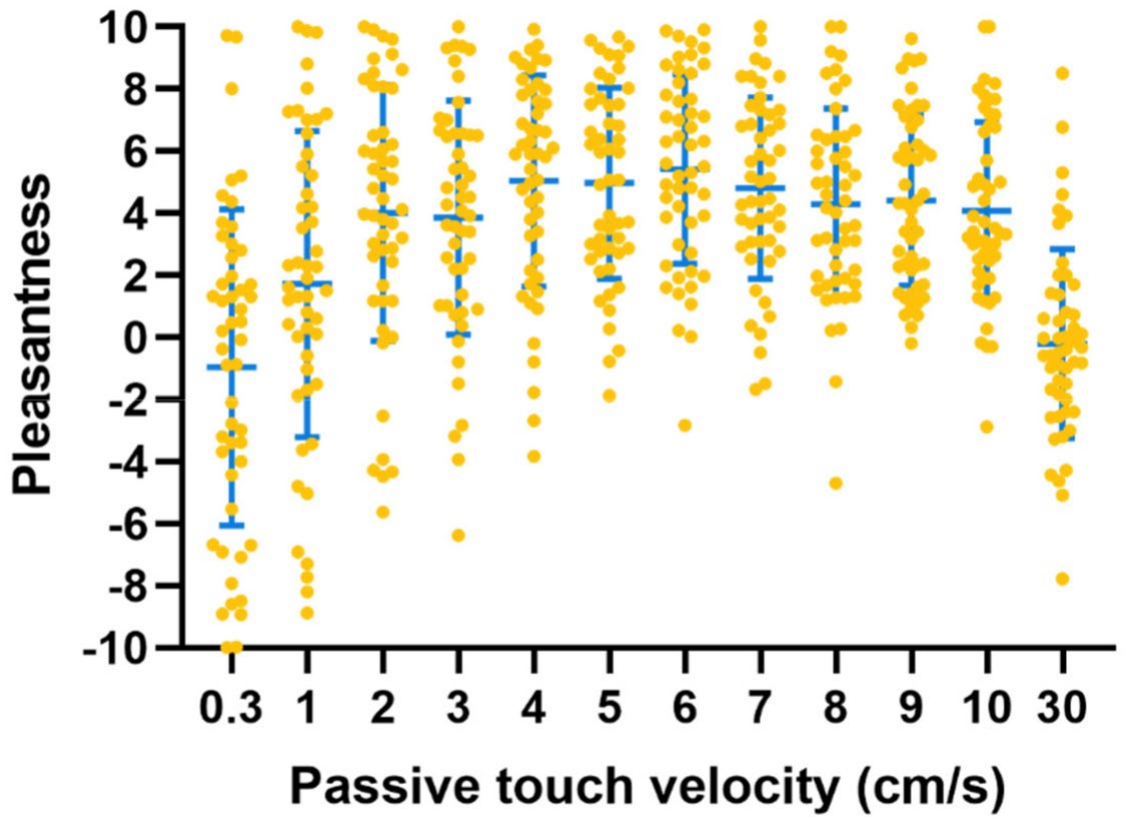
Pleasantness ratings for different touch velocities (passive touch). Higher values indicate higher pleasantness. Each orange dot represents the mean ratings of one participant per velocity. The blue bars indicate mean and standard deviation across participants.

Pleasantness ratings for each velocity averaged across repetitions per participant are shown in Figure 2.

**Figure 3:**
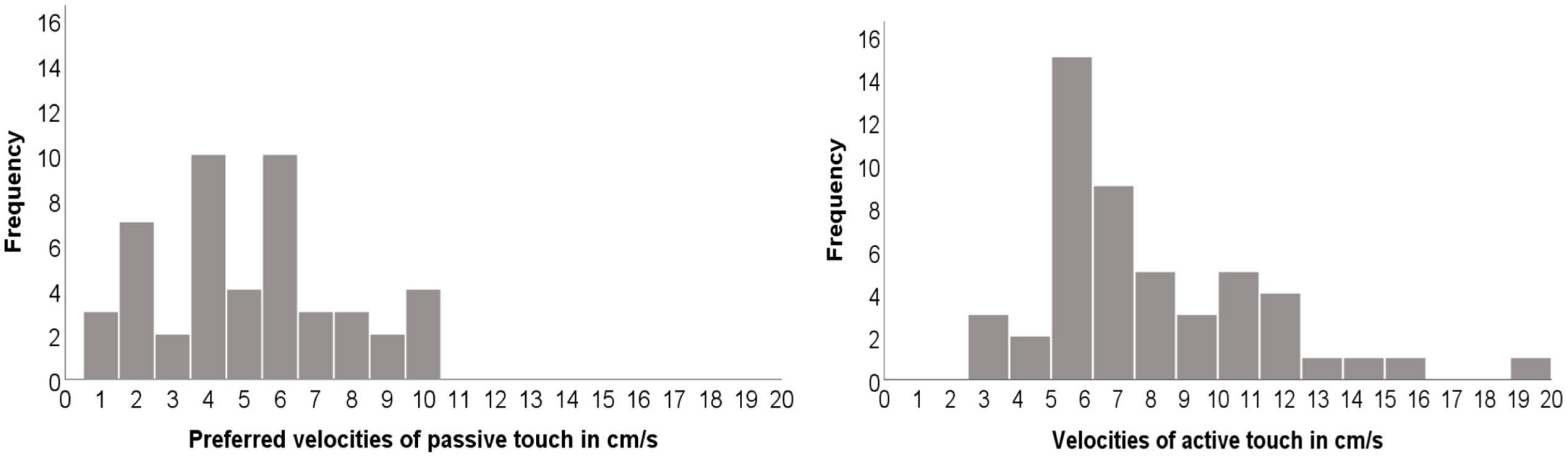
Distributions of preferred velocities of touch (left: passive touch; right: active touch).

When selecting the most preferred velocity per participant, there was considerable variation across participants. Nevertheless, there were peaks in the highest pleasantness ratings for the velocity of passive touch, corresponding to 4 and 6 cm/s (Figure 3, left panel), with an average of 5 cm/s (*SD* = 2.57). Similarly, the velocity used most frequently to perform stroking by the participants was 6 cm/s, with an average velocity of 7.8 cm/s (SD = 3.28) (Figure 3, right panel).

Passive and active touch preferences correlated moderately with each other (*r* = 0.35, *N* = 48, *p* = 0.01).

#### Preferred tempo of music

**Figure 4:**
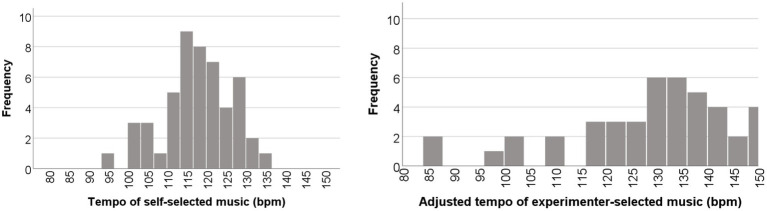
Distributions of preferred musical tempos (left: self-selected music; right: experimenter-selected music).

Across all participants, the most favored tempo of self-selected music had a mean of 117.59 bpm (SD = 8.94) and the most favored of the experimenter-selected music had a mean of 133.55 bpm (SD = 19.62). Note also that the standard deviation was higher for the experimenter-selected music, indicating higher uncertainty. Figure 4 illustrates the distributions for both of those measures.

#### Preferences for tempo of passive touch and self-selected music are related

As to the variation in tempo of the 5 self-selected music pieces within individuals, the standard deviation ranged from 7.08 to 33.71 bpm. Given the mean of 117.59 bpm across all individuals, the preferred velocity of self-selected music was thus fairly consistent across music pieces within individuals.

**Figure 5:**
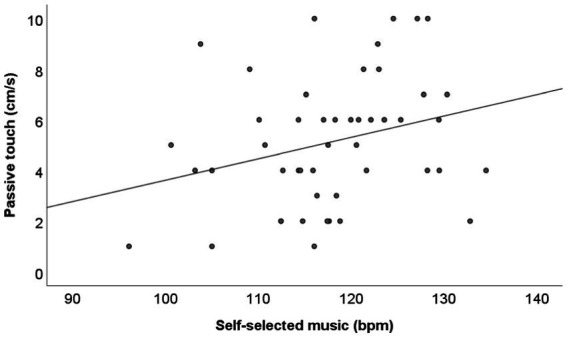
Passive touch (cm/s) against self-selected music (bpm) with regression line.

Passive touch and self-selected music correlated moderately with each other (*r* = 0.31, *N* = 48, *p* = 0.03; sig. With FDR = 0.25) (Figure 5). Passive touch did not correlate with experimenter-selected music (*r* = 0.05, *N* = 48, *p* = 0.76).

The preferred tempos for self-selected and experimenter-selected music were weakly correlated (*r* = 0.271, *N* = 50, *p* = 0.06). Active touch and experimenter-selected music were not correlated (*r* = 0.028, *N* = 50, *p* = 0.85). Self-selected music and active touch were weakly negatively correlated with each other (*r* = −0.26, *N* = 50, *p* = 0.07). Note that after multiple testing adjustment using the method of Benjamini and Hochberg both, the correlations between the preferred tempos for self-selected and experimenter-selected music and between self-selected music and active touch, will result in FDR-adjusted *p*-values <0.25.

#### Relationship between heart rate measurements and preferred tempos of touch and music

**Figure 6:**
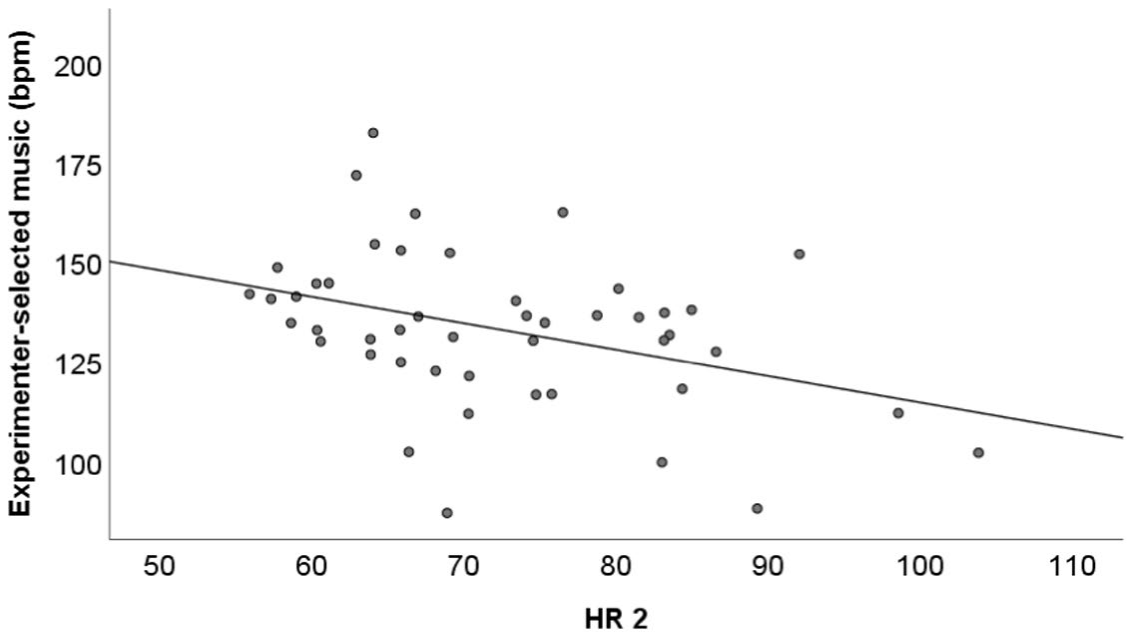
Experimenter-selected music against HR2 with regression line.

HR1 and passive touch approached a significant level of correlation (*r* = 0.284, *N* = 46, p = 0.06), but HR1 did not correlate with active touch (*r* = 0.03, *N* = 48, *p* = 0.85). HR2, however, showed a moderate negative correlation with experimenter-selected music (see Figure 6; *r* = −0.39, *N* = 47, *p* < 0.01, sig. With FDR = 0.25).

#### HRV increased while mean heart rate and both positive and negative mood decreased over the course of the experiment

Exploratory analysis showed that heart rate was significantly lower during the second measurement (HR2 *M =* 72.6, SD *=* 11.26) than during the first measurement (HR1 *M =* 78.05, SD *=* 13.6; t (45) = 6.29, *p* < 0.0001, sig. With FDR = 0.25, standardized mean difference d = 0.93)*.*

RMSSD was higher during the second measurement (RMSSD2 *M* = 49.44, SD = 26.09) than the first measurement (RMSSD1 *M* = 40.89, SD = 22.27; *t* (45) = −4.60, *p* < 0.0001, sig. With FDR = 0.25, *d* = 0.68). Both positive and negative mood decreased during the experiment (PANAS1 positive *M* = 29.96, SD = 5.55; PANAS2 positive *M* = 23.7, SD = 6.47; *t* (47) = 7.63, *p* < 0.0001, sig. With FDR = 0.25, *d* = 1.10; PANAS1 negative *M* = 13.09, SD = 3.54; PANAS2 negative *M* = 11.41, SD = 3.09; *t* (43) = 3.83, *p* < 0.0001, sig. With FDR = 0.25, *d* = 0.58).

#### Body awareness did not mediate the relationship between touch and music tempo preferences

Body awareness did not mediate the relationship between self-selected music and passive touch (*b* = −0.08, 95% CI [−0.41; 0.11]). There was only a significant direct effect of self-selected music on passive touch (*b* = 1.08; 95% CI [0.81; 2.09]). Self-selected music significantly predicted passive touch (*t* = 2.18; *p* = 0.03), explaining 10% of the variance in passive touch.

## Study 2: Observing repeated stroking and musical drumming (both by brushing)

In Study 1, it is likely that a host of factors, like the different musical genres, melodies and even text (i.e., lyrics in some of the self-selected music) may all have influenced preferences for a specific musical piece. Thus, we decided that in Study 2, only the rhythmic features of actual drum beats would be heard, but also seen in a set of videos. In addition, in the previous study, the passive touch was based on single, discrete, strokes, the velocities of which were compared with ongoing music. In study two, the tactile and auditory tempos were directly comparable to each other as the touch was presented in the form of video examples of continuous and repeated touch stimulations by stroking on the underarm back and forth.

Furthermore, in Study 1 the tempos presented as alternatives for preferred touch and music had different ranges, as the fastest touch velocities of 10 and 30 cm/s correspond to approximately 60 and 180 strokes per minute, whereas the lowest preferred tempo for self-selected music was 96 bpm and the highest was 136. In Study 2, we presented touch velocities that overlapped some of the preferred values found for self-selected music, as found in Study 1.

Finally, we assessed the musicality of the participants, as this could be associated with the ability to synchronize touching behavior with preferred music (Rajendran et al., 2018).

Due to the ongoing Covid-19 pandemic at the time of the study that prohibited access to the lab, the experiment could only be conducted as an online-study, and instead of actual touch, participants rated the touch they observed another person receiving. This choice was also supported by several studies showing that vicarious stroking touch can be evaluated in a way similar to actual stroking touch (Morrison et al., 2011; Walker et al., 2017; Bellard et al., 2022).

### Materials and methods

Prolific^2^ was used as a platform for recruitment. The videos and questionnaires were presented *via* the University of Oslo’s web site for internet surveys.^3^ The study obtained approval from the Norwegian Centre for Research Data/.^4^

#### Participants

In study 1, the correlation was of medium effect size (*r* = 0.3). To account for potentially smaller effects for the online study, also given the complicating factor of using a third modality mediating the tactile stimulation in a ‘virtual mode’, we decided to perform statistical power analyses based on the sample required for a small effect size of 0.2, but aiming for a power of 0.8 and statistical significance level 0.05 using GPower version 3.1.9.7. (Faul et al., 2009). The underlying assumption which was tested was that Pearson’s correlation would be equal to zero. Hence, N = 191 participants were determined to be appropriate. To account for potential invalid datasets (e.g., incomplete responses), 200 English-speaking participants from any country in an age range between 18 and 55 were recruited.

The data of all participants were screened first for consistency, based on the answer to a control question in an initial catch trial, and the absence of variation in the answers given (always giving the same answer). One participant that provided contradictory answers in one of the questionnaires (Goldsmiths Musical Sophistication Index; Mullensiefen et al., 2014) was contacted and repeated the study. As the answers from the second round differed widely from the first round in all tasks, we had to exclude this participant from the analyses. Thus, the final sample consisted of 199 participants (mean age M = 27, SD = 7.7), 87 women and 112 men. About half (48%) reported to be employed or self-employed, 44% reported to be students at a school or university, and 8% reported “other” occupation. 17 participants (8.5%) reported to take medication(s) for a mental health condition, of which antidepressants were named 16 times, anxiolytics twice, and benzodiazepines twice.

All 200 participants were compensated with an average amount of GBP 10 per hour.

#### Procedure

**Figure 7:**
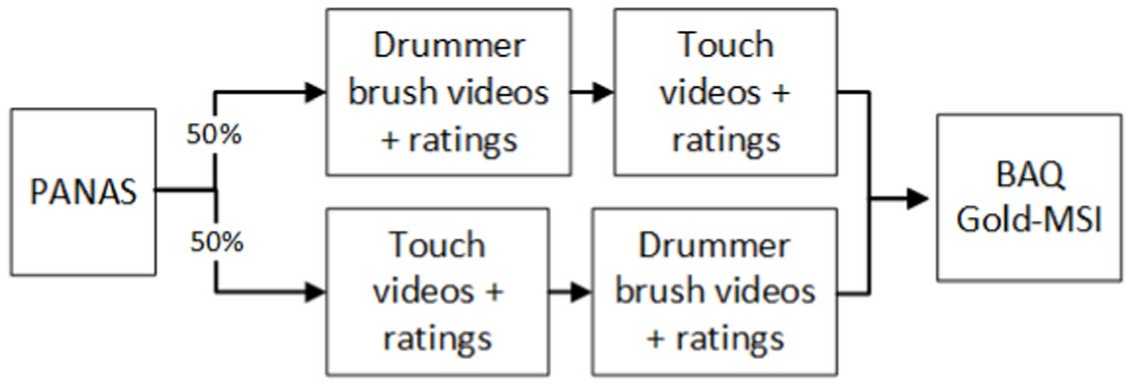
Order of tasks and measures in experiment 2.

All participants started out with filling in the mood questionnaire PANAS (see Figure 7).

**Figure 8:**
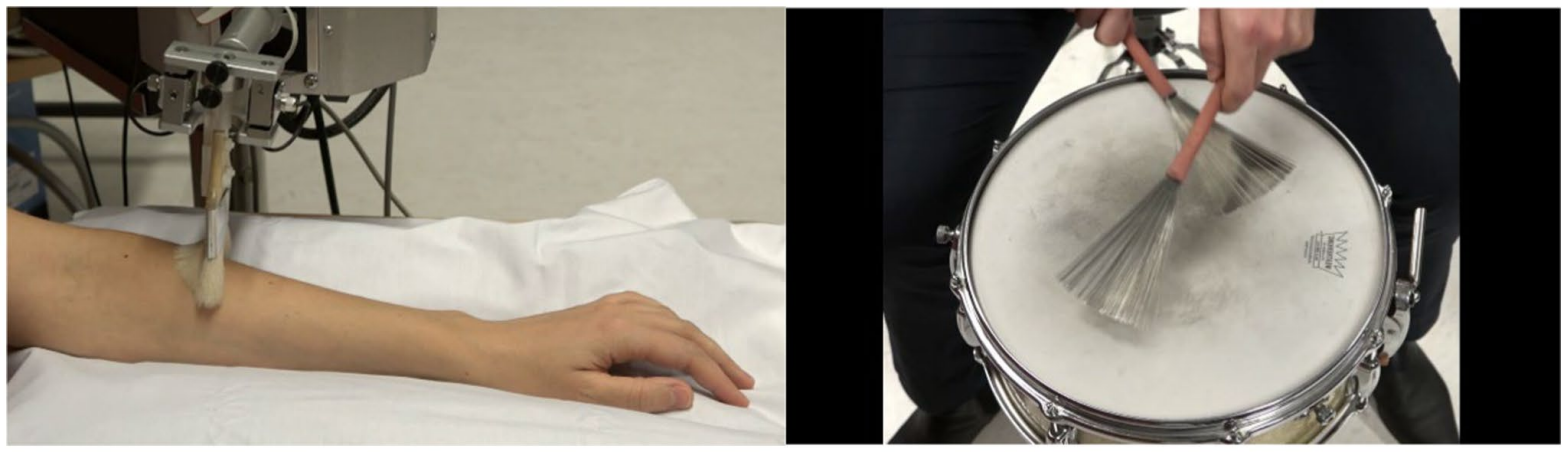
Still frames from videos of ‘arm brushing (left) and ‘drum brushing’ (right) conditions.

Participants then watched 12 videos of an underarm stroked across a length of 10 cm by a painter’s brush held by the robotic device (Figure 8 left) used in study 1 (RTS, Dancer Design). Observed stroking is typically rated similar to experienced stroking when two or three velocities are presented (Morrison et al., 2011; Walker et al., 2017; Bellard et al., 2022). The velocities presented were the same 12 as in study 1 (0.3, 1, 2, 3, 4, 5, 6, 7, 8, 9, 10 and 30 cm/s), and the additional velocities of 16, 19, 20, 21, 22. These additional velocities were introduced as the respective number of strokes per minute would approach the number of beats per minute of some presented drummer rhythms. For example, a stroke of 16 cm/s across the 10 cm distance will take 0,625 s, thus amounting to 96 strokes per minute.

Following each touch observation, participants answered two questions on a 13-point Likert scale from −6 to +6. The first question was: “Rate how pleasant you think the touch feels to the person in the video” (similar to the wording used by Morrison et al., 2011). In addition to the numbers, the endpoints of the scale were marked as “pleasant” and “unpleasant.” The second question was: “How much would you like to be touched like that?” with the endpoints “very much” and “not at all.”

Participants also watched short video sequences of a professional drummer using a drum brush with 12 different tempos. The films showed the drummer’s hands holding two drummer brushes from above (Figure 8) while he played a standard ‘jazz swing’ brushing pattern. The tempos were derived from the normal distribution of the preferred tempos from study 1, where the lowest and highest tempos preferred were 96 and 136. Within the range of 96 and 136, we selected further values that followed a symmetrical unimodal distribution to result in a higher frequency of values in the middle. In total, the tempos 96, 101, 106, 111, 113, 115, 117, 119, 121, 126, 131, 136 bpm were used. Here, the distance between the first 4 and last 4 tempos is 5 (oriented at half the SD from study 1, which is 4.35), and the distance between the tempos in the middle is 2 (oriented at a fourth the SD from study 1, which is 2.2). Following each video, the participants answered the question “How pleasant was the rhythm?” on a 13-point Likert scale from −6 to +6. In addition to the numbers, the endpoints of the scale were marked as “pleasant” and “unpleasant.”

These videos of arm and drum brushing were presented in two separate blocks, the order of which was counter-balanced across participants. Within each block, the different tempo velocities were presented in three different pseudo-randomized orders to the participants.

Following rating the videos, participants filled in the BAQ (Shields et al., 1989) and the Goldsmiths Musical Sophistication Index (Gold-MSI; Mullensiefen et al., 2014). The Gold-MSI assesses self-reported musical skills and behaviors on multiple dimensions, and also identifies a general factor of musical sophistication that arises from the correlations between these dimensions.

#### Measures

All data were analyzed with R version 4.2.1 (R Core Team, 2022).

First, we attempted to determine the most pleasant velocity as the one receiving the highest pleasantness rating, as was done in Study 1. However, this turned out to not be possible, as the majority of participants gave the highest rating to more than one velocity for both arm and drum brushing (see Results).

Since the ratings of each individual did not develop linearly across arm brushing velocities or drum brushing tempo, the data were fit with a mixed model based on natural cubic splines. A basis matrix with two (drum brushing) or three (arm brushing) degrees of freedom for representing the family of piecewise-cubic splines was fitted to the brushing velocity data and the resulting basis spline functions were included alongside an intercept in a linear mixed models both random and fixed effects to explain drum brushing tempo. Individual courses of the ratings across different arm brushing velocities and drum brushing tempos were estimated (based on the random effects), and the mean curve across all participants (using the fixed effect estimates). Conditional and marginal R-squared measures of explained variation for mixed-effects models were used to assess model fit (Nakagawa et al., 2017).

To determine if there were groups of participants with similar preferences (curve shapes), two hierarchical cluster analyses with complete linkage were performed with different distance metrics. Firstly, participants were clustered based on the Pearson’s correlation of the fitted curves, and secondly, based on the Euclidean distance of the mean values. To determine the appropriate number of clusters, the average silhouette width was calculated from among 2 to 9 clusters.

To find out whether preferences for the tempos of arm brushing and drum brushing were related, we assumed that individuals within a certain cluster for arm brushing should also be in one cluster for drum brushing. Pearson’s Chi-squared test was performed to compare the number of participants within each touch cluster that were also grouped within one and the same drum cluster. This was done separately for the two clustering distance metrics.

In case of an association, we had originally intended to investigate if it was influenced by body awareness, musical sophistication, or positive or negative affect. However, since no such association was observed (see Results), the questionnaires were not followed up.

### Results

#### Touch liking is velocity-dependent

**Figure 9:**
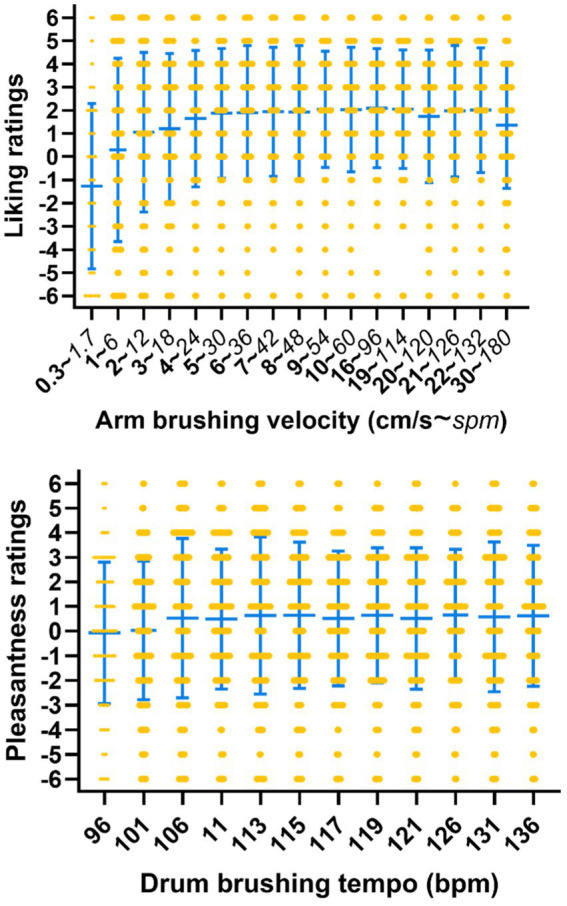
Liking ratings for different arm brushing velocities (upper) in cm/s or right above in strokes per minute: spm, one dot per participant. Pleasantness ratings (lower) for different drum brushing tempos, one dot per participant. Higher values indicate higher liking and higher pleasantness. Blue bars indicate mean and standard deviation across participants.

Liking ratings per velocity and participant are shown in Figure 9 (upper). Visual inspection indicates that liking was highest for the middle velocities.

#### Drum brushing preferences

The slowest tempos appeared to be slightly less pleasant than the higher tempos. Pleasantness ratings for each drum brushing tempo are shown in Figure 9, lower panel.

#### No clear preferences for one particular velocity of arm and drum brushing

In Study 1, the favorite velocity of each individual was determined by picking the arm brushing velocity that had received the highest mean rating. In study 2, however, it turned out that the highest rating was often given to more than one velocity. In response to the question “pleasantness for the other,” the highest rating (+6) was given to one velocity by 67 participants, to two velocities by 47 participants, and to more than two velocities by 88 participants. Similarly, in the question “liking for oneself,” the highest rating was given to one velocity by 67 participants, to two velocities by 40, and to more than two velocities by 91 participants. In addition, in participants who gave the highest rating to two velocities, these velocities appeared to be often far apart from each other (e.g., 22 cm/s corresponding to 132 spm, and 6 cm/s corresponding to 36 spm). Thus, differently from being physically touched, no clear preferences emerged in this observation and online paradigm, and it was not possible to uncontroversially determine a preferred tempo.

Regarding preferred drum brushing, the highest rating was given to one tempo by 95 participants, to two tempos by 44, and to more than two by 59. Thus, a similar picture as for the touch ratings emerged for the drum brushing beats, although a larger number of participants named one preference than in the other condition.

**Figure 10:**
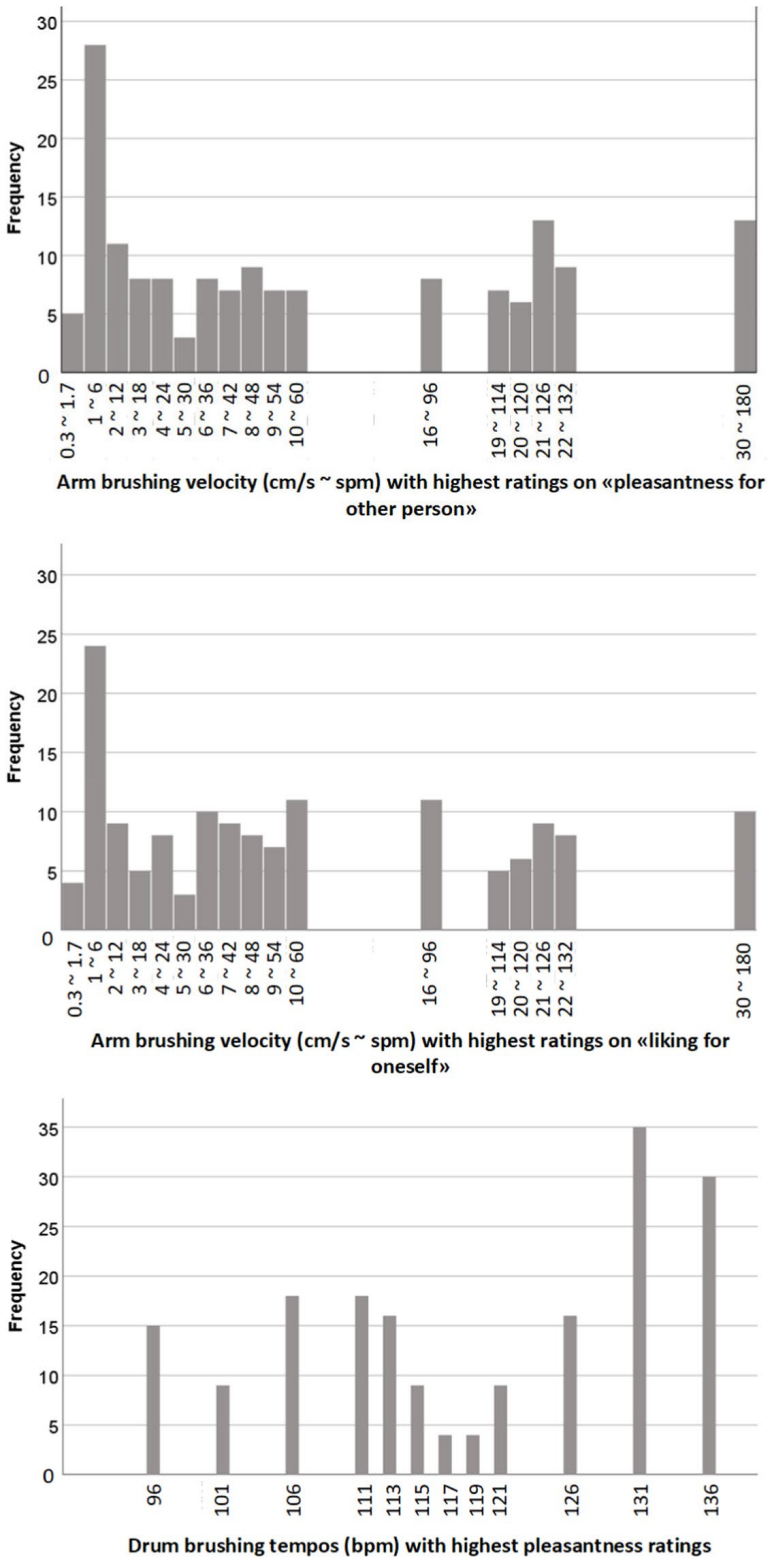
Histograms of the distributions of preferred arm brushing and drum brushing tempos for participants who gave two or fewer highest ratings. Upper: most pleasant velocity for other person (*N* = 114); middle: most liked velocity for oneself (*N* = 107), lower: most pleasant drum brushing tempo (*N* = 139).

Figure 10 shows the distribution of ratings for participants who gave the highest rating to two or fewer tempos.

#### Highly variable ratings patterns for tempos of observed arm and drum brushing

**Figure 11:**
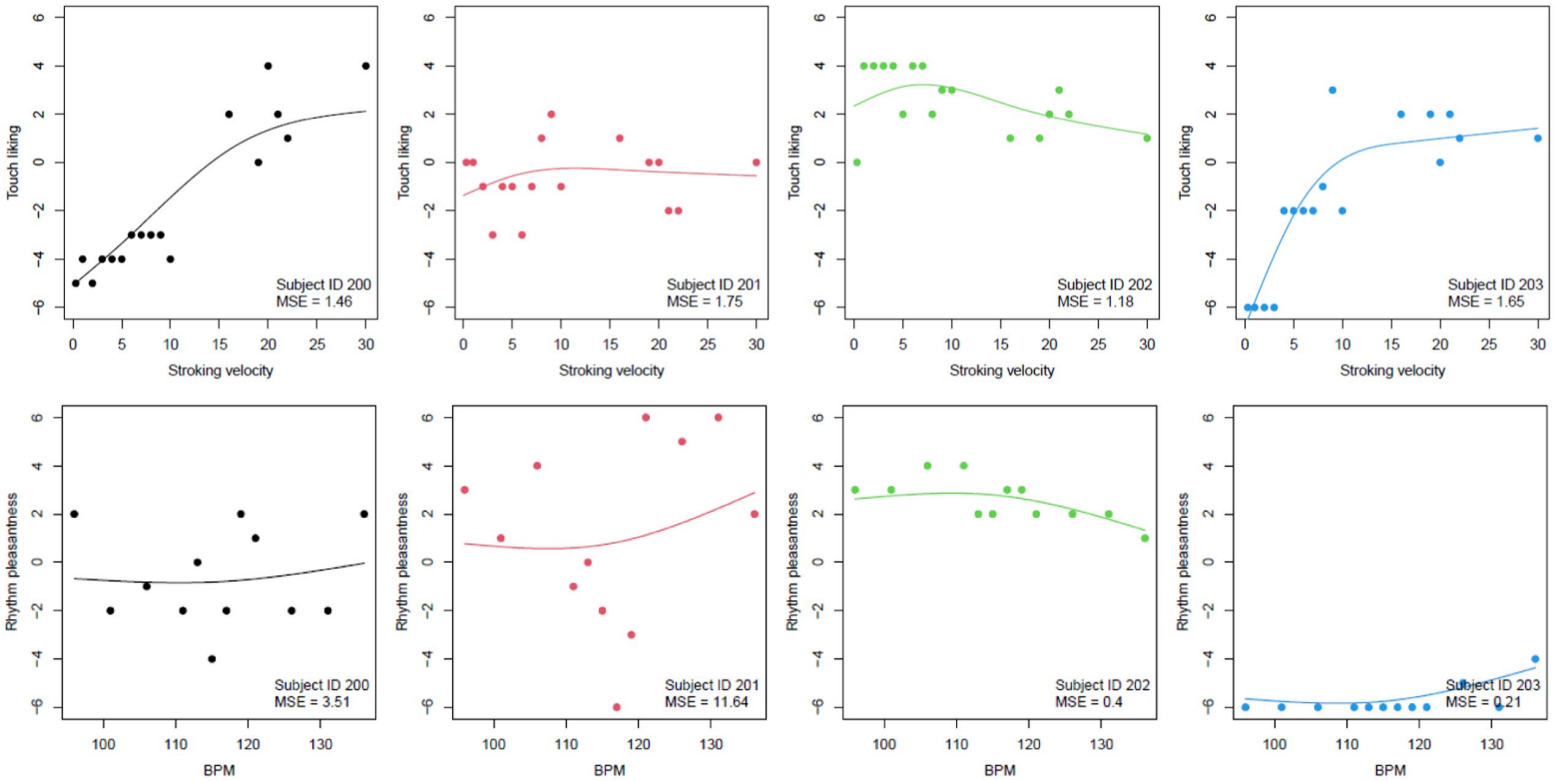
Touch (upper) and beat rating patterns (lower) with fit line for four participants. MSE = mean of the squared residuals as a measure of the goodness of the fit.

**Figure 12:**
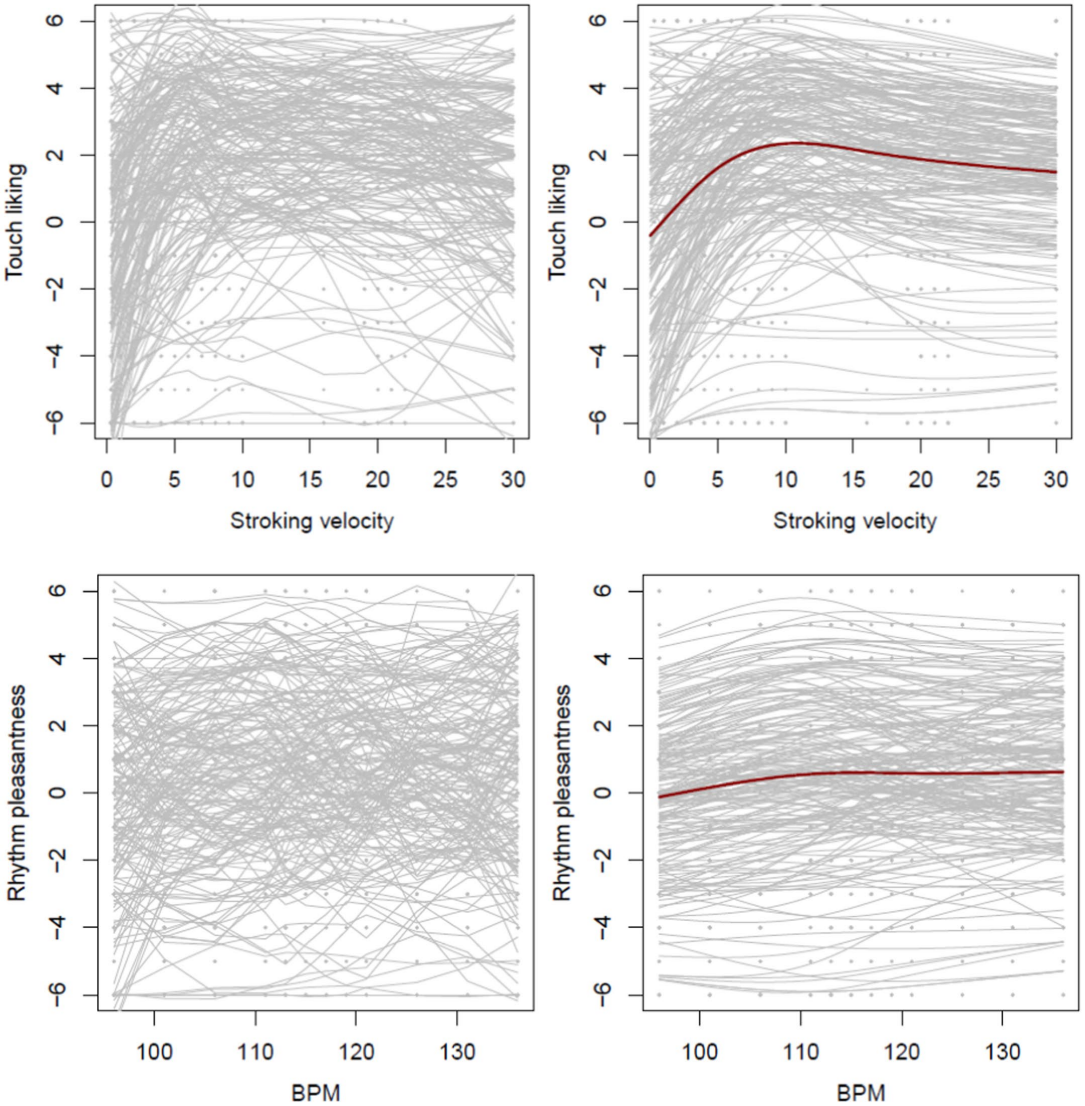
Individual curves for touch (upper) and beats (lower). The left column shows curved as observed in the data (loess-smoothed), and the right column shows curves as estimated with the model (right). The mean estimate across all participants is displayed in dark red.

Participants showed very different responses to arm and drum brushing across different velocities (Figures 11, 12). Model fits were assessed by conditional and marginal R-squared measures of explained variation for mixed-effects models. The marginal R-squared values, which only include the fixed effects, were 0.061 and 0.005, respectively, which shows that the global mean curves (Figure 12, right column) do a very poor job in explaining the variability in the data. This highlights the large heterogeneity in participant responses which is also illustrated in Figures 11, 12. The conditional R-squared values, which include the random effects and thus reflect the average model fit of the individual curves fitted to each participant, were 0.726 and 0.671 for the arm brushing and drum brushing models, respectively, which indicates a very good model fit for individual participants.

The right plot includes the estimated mean curve across all participants based on the estimated fixed effects and shows an increase in the touch liking score until a stroking velocity of about 12, and then a decline. The mean curve for beats is rather flat but with a slight continuous increase across the range of BPM values.

#### Participants can be grouped according to their preferences within each modality

**Figure 13:**
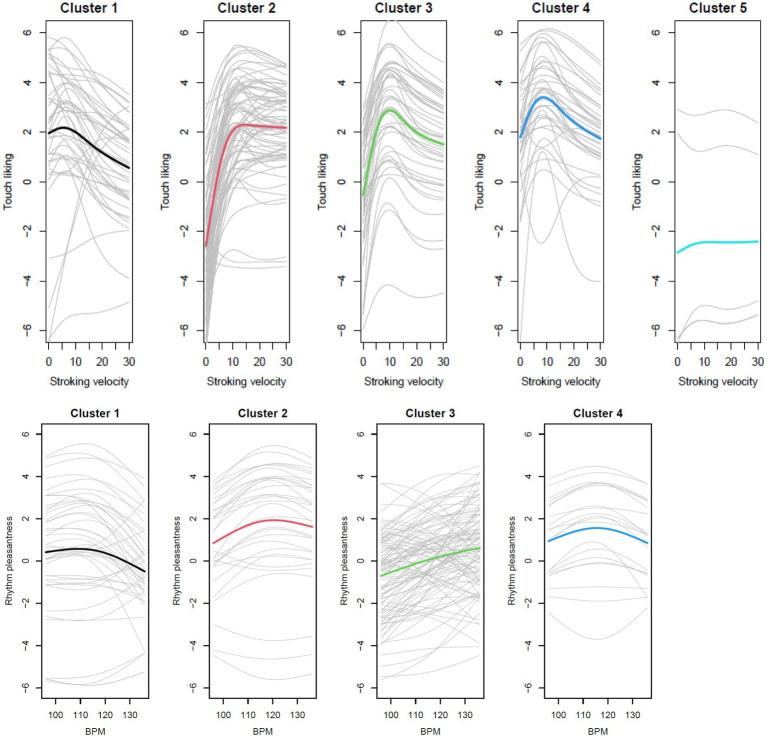
Clustering identified five different curve forms for touch ratings (upper) and two different curve forms for beat ratings (lower).

When clustering was performed according to the similarity of curve forms, the average silhouette width suggested 5 clusters for touch ratings (Figure 13, upper row) and 4 for the beat ratings (Figure 13, lower row).

The five touch rating clusters differ in starting points, in the relative increase of pleasantness from the lowest to the middle velocities, and the change from the middle to the highest velocities. The participants in cluster 5 showed a rather flat curve throughout. The two beat rating clusters present a flatter curve, where cluster 1 shows a very small but steady increase of pleasantness with music beat, where cluster 1 shows a slight preference for the middle beats.

**Figure 14:**
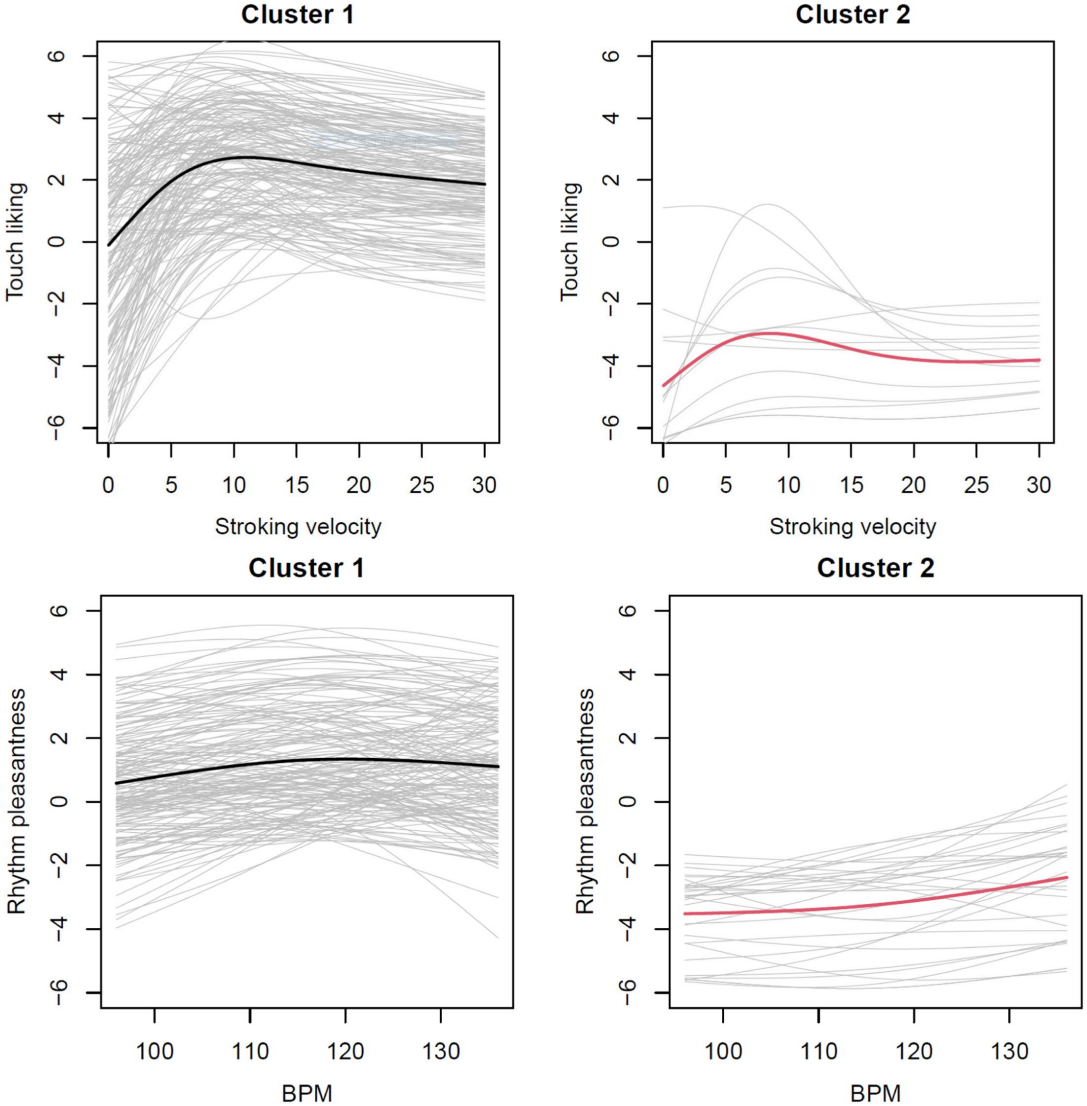
Clustering identified three different mean groups for touch ratings (upper) and two different mean groups for beat ratings (lower).

When clustering was performed according to the mean values in each curve, the average silhouette width suggested 2 clusters for touch ratings (Figure 14, upper row) and 2 for the beat ratings (Figure 14, lower row).

Participants in touch cluster 1 disliked the slow velocities but liked velocities from 3 cm/s and over, whereas participants in touch cluster 2 rated all velocities as pleasant, and the middle velocities as most pleasant. Participants in touch cluster 3 disliked touch at all velocities, with the smallest dislike for the middle velocities. For the beat clusters, participants in cluster 1 showed a very slight increase in pleasantness until ~110 bpm and then a plateau, with the average ratings slightly pleasant. In contrast, participants in cluster 2 rated all music beats as unpleasant, but with an increase in pleasantness for the faster tempos.

We assumed that similar preferences for touch velocities and music beats would be evident in the fact that participants who are in a cluster for touch velocities are also in one and the same cluster for music beats (see Table 1). However, the number of participants in the same clusters for both touch and beats was not different when clusters were based on the curve forms (Chi-squared = 14.069; df = 12, *p* = 0.296). Also, for the clusters based on the means, participants from one touch cluster were usually not in the same beat cluster (Chi-squared = 0.141, df = 1, *p* = 0.707). Thus, there was no evidence for the individual preferences for touch velocities and music beats being similar.

**Table 1:** Number of participants in one touch cluster that were in one and the same beat cluster.

		Curve form clusters			
		Beat cluster			
		1	2	3	4
Touch cluster	1	10	2	22	8
	2	18	12	37	6
	3	8	8	24	3
	4	5	7	21	3
	5	2	2	1	0

		Mean clusters	
		Beat cluster	
		1	2
Touch cluster	1	158	2
	2	10	3

### Discussion

Two separate studies investigated if the preferred velocity or tempo when listening to music and the velocities at which people would like to be touched (either directly or indirectly from vision) are related. Whereas study 1 found such a relationship, this was not obvious from study 2. Thus, no conclusions can be drawn either for or against the idea that tempo in music is related to tactile stimulation.

#### Preferred touch and music tempo in relation to the literature

In Study 1, the stroking velocities that participants enjoyed most were 4 and 6 cm/s. Indeed, 6 cm/s was also the velocity the participants used themselves to stroke the fur. This fits with previous findings showing that individuals tend to touch their partners with velocities similar to those at which they would like to be touched themselves (Triscoli et al., 2017a). Specifically, our participants showed a preferred velocity for receiving touch at a mean of 5.12 cm/s and for giving touch at a mean of 7.81 cm/s. These velocity preferences for both passive and active touch correspond to the range of velocities that optimally activate CT fibers, namely between 1 cm/s and 10 cm/s (Löken et al., 2009).

In Study 2, participants did not appear to have any clear preferences for a particular stroking velocity. This could be because the participants did not actually feel the touch, but only observed it. This suggests that imagining how an observed touch may feel is not perfectly reliable when a high number of alternatives are provided. However, across all participants, the ratings showed the typically observed rating pattern for experienced touch with lower pleasantness ratings at the slowest and fastest velocities (Croy et al., 2020). Thus, participants clearly paid attention to the task and answered in a differentiated way.

In Study 1, the preferred tempo of music among our participants peaked at 117.6 bpm for self-selected music pieces, which corresponds to previous findings in the literature where preferences of 100–120 bpm were reported (Moelants, 2002; Etani et al., 2018). The adjusted tempo for experimenter-selected music appeared to be somewhat higher, at 133.6 bpm. Future research might clarify if an increase in tempo for unfamiliar music is a reliable finding and, if so, the reasons for such an acceleration in tempo.

In Study 2, most participants had no clear preference for one particular tempo. In those individuals who named only one or two most pleasant tempos, the two highest tempos of 131 and 136 bpm dominated. This is different to study 1, where the highest tempos were the extremes of a normal distribution. This may be due to the mode of presentation and the similarity of the stimulus material within each category, with the only difference being the tempo of the drum brushes. On average across all participants, the two slowest tempos were rated as less pleasant than the faster tempos.

Thus, although the preferences for touch and music observed in the current study are consistent with the literature, they are clearly dependent on the measurement mode.

#### Inconclusive relationship between preferred tempos for touch and music

Whereas study 1 found a weak relationship between preferred velocity of received touch and music tempo, study 2 found no such relationship.

This difference may be due to the use of different methods to measure the preferred tempo of touches and music. In study 1, the preferred tempo was estimated implicitly based on music selected by the participants. In study 2, the preferred tempo was collected explicitly by judging musical beats stripped from other musical aspects (e.g., melody or harmony). Furthermore, there are also differences in the continuousness or discreteness of the stimuli used. In study 1, the music pieces were continuous, whereas there was a break after each brush stroke for the participants to give their rating. Nevertheless, this difference did not interfere with revealing a commonality between the timing of the arm brushing and those of musical pieces. The fact that we found a correlation in study 1 may imply that the discrete or continuous aspect of the touching motion is not crucial for a shared or overarching timing processor or pattern generator between modalities.

In contrast, in study 2 brushing as used musically in jazz was semi-continuous both in terms of bodily motion and acoustic feature compared to the discrete, percussive, beats that can be heard in most music. Hence a beat detection device in the brain may find it harder to extract the beat from ‘sliding’ sound actions. Yet, the brushing, even when continuous (back and forth) has salient temporal points corresponding to the inversions of direction. Still, no communality between arm brushing and drum brushing could be found. One reason may be that a beat detection device in the brain may find harder to extract the beats from two ‘sliding’ sound actions (as in Study 2) than when at least one of them has clearly defined beat inter-onset-intervals (as the music in Study 1).

Another reason could be that participants in study 2 rated tactile stimulations vicariously, by watching the arm of another unknown person being touched in a video. This led to less pronounced preferences for touch compared to the “real” application. Thus, the different modes of touch presentation could also explain why the relationship between touch and music differed in the two studies.

#### Potential underlying mechanisms for a relationship

The fact that participants in study 1 who preferred faster music also preferred faster touch may indicate that the processing in the two areas is similar. Such similarity may be achieved by an integration between the rhythms of audition and touch in shared cerebral systems (Cameron et al., 2022; Ito et al., 2022) or *via* a central pattern generator (Marder, 2001; Dietz, 2003; Styns et al., 2007). Humans tend to prefer similar tempo for music and spontaneous walking speed (Schneider et al., 2010), namely around 120 steps per minute (Pailhous and Bonnard, 1992; MacDougall and Moore, 2005) for walking and around 120 bpm for music (Moelants, 2002; Etani et al., 2018). Interestingly, this also turned out to be the preferred musical tempo of self-selected music in Study 1. Indeed, whereas these previous studies showed that music tempo is related to walking and tapping speed, our data suggest that music tempo may also be related to the velocity of motion with one would like to be touched. However, this relationship was only present for self-selected music, not for experimenter-selected music.

#### Experimenter-selected music and preferred touch velocity were not related

Passive touch and active touch did not correlate with experimenter-selected music. One reason for this could be that the pieces chosen for adjusting music tempo were all from the genres of house music and jazz. Although the music pieces themselves were unknown to the participants, the styles might have primed a certain standard velocity that is typical for the genre. In contrast, the individually chosen music pieces for self-selected music stemmed from a much larger range of musical styles. Alternatively, unfamiliar music (and likely different from the participant’s preferred genre) might not recruit the same “pacemaker” equally effectively.

There are also differences in the way active touch and experimenter-selected music were measured: for experimenter-selected music, the final value with which the participant was satisfied was recorded, whereas for active touch, the mean value was used. As there presumably is a dynamic in the adjustment of both music tempo and stroking velocity, this difference may have masked a possible relationship between the two measures.

#### The role of arousal

It has been suggested that also the cardiovascular system is controlled *via* a central pacemaker in the brain (Julien, 2006; Pfurtscheller et al., 2017; e.g., Verberne and Owens, 1998) and that cortical functions in turn are modulated by the heartbeat (Lacey, 1970; Mather and Thayer, 2018). In the present study, heart rate did not correlate with stroking velocity preference (passive touch velocity). This may have several reasons, the most likely one being that there were no heart rate measurements close in time, as the self-selected musical pieces were not collected on the day of the experiment, but earlier.

In contrast to passive touch velocity, active touch velocity moderately correlated with the adjusted tempo of the experimenter-selected music, but only when measured at the second time point in study 1. This time point was after the active and passive touch tasks, but before adjusting experimenter-selected music. This indicates on the one hand, that heart rate did not influence touch perception or behavior, and on the other hand, that instead touch may have influenced heart beat. This is in line of studies showing that holding a dog (Straatman et al., 1997), actively stroking a horse (HAMA et al., 1996) or a breathing animal-like robot (Sefidgar et al., 2016) can reduce heart rate, although this effect was not found for stroking the own partner (Triscoli et al., 2017a).

The correlation at the second time point indicated that participants with higher arousal tended to adjust the presented music pieces to lower tempos. At first, this appears to be in contrast to studies on mothers stroking their children, where higher heart rates were associated with faster – not slower - stroking velocities (Van Puyvelde et al., 2019; Bytomski et al., 2020). However, the motives may be different in these two situations. One motive of giving touch is to modulate the receiver’s mood or arousal, e.g., to calm down (Jones and Yarbrough, 1985). In the present study, the receiver was a heated fur pillow, and the instruction was “as if they were stroking a person close to them,” thus, without any communicative or mood-regulating intent (e.g., to comfort, to soothe). Instead, the adjustment of experimenter-selected music may have served the aim of regulating the arousal regulation of the participant, by searching for calming stimuli when HR is high. Along these lines, it has been proposed that a situation that requires more arousal would promote the preference for stimulating music (North and Hargreaves, 2008).

The higher correlation between the adjusted tempo of experimenter-selected music and HR during the second than the first measurement might also be related to the higher HRV during this later measurement. HRV (RMSSD), a measure reflecting parasympathetic adaption capacity (Shaffer et al., 2014), increased after touch had been administered. This is consistent with previous findings of increasing HRV during CT-optimal touch (Triscoli et al., 2017b; Van Puyvelde et al., 2019), although participants in the present study were also touched at non-CT-optimal velocities. The increased HRV may be due to the tactile stimulation or to adaptation to the experimental setting.

#### Limitations and outlook

The usefulness of the average tempo of participant’s freely chosen self-selected music, which was used as a measure of individual music tempo preference in Study 1, needs to be validated in further studies. Possibly, this measure might also be adjusted by including a larger number of musical pieces collected over several days. Nevertheless, the present average measure appeared to capture some qualities that define both musical and tactile preferences.

Although the results from study 1 may support related preferences between touch and music, study 2 did not. This suggests that the effects do not occur in any situation, but that they are dependent on the experience, especially whether directly sensing on the body is involved and for both types of stimulations. Future research should further probe the relationship between touch and music beat by investigating whether changes in the preferences for one modality alter the preferences in the other modality.

## Data availability statement

The raw data supporting the conclusions of this article will be made available by the authors, without undue reservation.

## Ethics statement

The studies involving human participants were reviewed and approved by the ethical committee at the Department of Psychology, University of Oslo. The patients/participants provided their written informed consent to participate in this study.

## Author contributions

US and BL conceived and designed the studies and gained ethical approval. US analyzed the data of study 1 and MZ analyzed the data of study 2. US wrote the manuscript draft. All authors contributed to the article and approved the submitted version.

## Funding

The study was supported by the Norwegian Research Council *via* the Centre of Excellence program (RITMO 262762). US was also supported by ERA-NET-NEURON, JTC 2020/Norwegian Research Council (grant number 323047).

## Conflict of interest

The authors declare that the research was conducted in the absence of any commercial or financial relationships that could be construed as a potential conflict of interest.

## Publisher’s note

All claims expressed in this article are solely those of the authors and do not necessarily represent those of their affiliated organizations, or those of the publisher, the editors and the reviewers. Any product that may be evaluated in this article, or claim that may be made by its manufacturer, is not guaranteed or endorsed by the publisher.

## Footnotes

^1^ https://soundcloud.com/co-pilot/self-image

^2^ www.prolific.ac

^3^ nettskjema.uio.no

^4^ https://www.nsd.no/en
